# Serendipitous Offline Learning in a Neuromorphic Robot

**DOI:** 10.3389/fnbot.2016.00001

**Published:** 2016-02-15

**Authors:** Terrence C. Stewart, Ashley Kleinhans, Andrew Mundy, Jörg Conradt

**Affiliations:** ^1^Centre for Theoretical Neuroscience, University of Waterloo, Waterloo, ON, Canada; ^2^Mobile Intelligent Autonomous Systems Group, Council for Scientific and Industrial Research, Pretoria, South Africa; ^3^School of Computer Science, University of Manchester, Manchester, UK; ^4^Department of Electrical and Computer Engineering, Technische Universität München, München, Germany

**Keywords:** adaptive systems, mobile robotics, neurocontrollers, neuromorphics, robot control

## Abstract

We demonstrate a hybrid neuromorphic learning paradigm that learns complex sensorimotor mappings based on a small set of hard-coded reflex behaviors. A mobile robot is first controlled by a basic set of reflexive hand-designed behaviors. All sensor data is provided via a spike-based silicon retina camera (eDVS), and all control is implemented via spiking neurons simulated on neuromorphic hardware (SpiNNaker). Given this control system, the robot is capable of simple obstacle avoidance and random exploration. To train the robot to perform more complex tasks, we observe the robot and find instances where the robot accidentally performs the desired action. Data recorded from the robot during these times is then used to update the neural control system, increasing the likelihood of the robot performing that task in the future, given a similar sensor state. As an example application of this general-purpose method of training, we demonstrate the robot learning to respond to novel sensory stimuli (a mirror) by turning right if it is present at an intersection, and otherwise turning left. In general, this system can learn arbitrary relations between sensory input and motor behavior.

## 1 Introduction

A long-standing dream for robotics is to provide the same sort of intelligent, adaptive, and flexible behavior that is seen in living biological systems. Furthermore, by creating systems that emulate biological adaptivity, we can investigate intelligence in a very broad sense, including capabilities that are not yet seen in current machine learning methods (McFarland and Bösser, 1993). However, it is still an open research question as to how to use biological inspiration to construct improved methods of robotic control. There are few examples of neural networks being used for control (Janglová, 2005) in a robotic domain (Conradt et al., 2000). Typically, the hardware used is standard general-purpose computing, and the algorithms are machine-learning based. This means that, while they may take some high-level inspiration from biology, the algorithms themselves do not directly map to biological details.

Over the last few years, a bridge has been forming between neuroscience and robotics (Krichmar and Wagatsuma, 2011). Ongoing developments in neuromorphic hardware design now provide novel computing resources that are more suitable to directly implementing the types of computations found in biological systems, thus facilitating the use of observations and data provided by the neuroscience community. Practically speaking, we can now implement large numbers of neurons on a very small power budget, making neural control a promising energy-efficient direction for mobile robot applications. For example, IBM’s TrueNorth chip implements neural network algorithms at one 10,000th of the energy cost of traditional computers (Merolla et al., 2014). Ideally, this sort of hardware could be used to provide flexible adaptive control of systems while staying within a limited power budget.

To exploit this hardware, we propose a hybrid method combining a small set of simple explicit programed behaviors with a training phase for shaping robot behavior as desired. That is, we start with low-level hand-designed reflexive actions, such as backing up when a collision sensor is triggered. While these can be defined using any standard robot control methodology, here, we implement these simple rule-based behaviors using neuromorphic hardware. Of course, these basic behaviors are extremely limited, and it is difficult to hand-design more complex actions, especially if those actions depend on complex environment-dependent stimuli. In the final example demonstrated in this paper, we want the robot to turn left or right depending on whether or not it is currently facing a mirror. Rather than attempting to hand-design a complex reasoning algorithm about detecting mirrors, we instead only hand-design the simple reflexive behavior of collision avoidance. The goal of this research is to then use a separate training phase where the robot can learn this more complex task. Rather than programing this task, the idea is to simply manually identify situations where the robot does what we wanted it to do *by accident* and use those situations as the basis for training.

Our study looks to demonstrate this flexible control system using neuromorphic hardware and neural-based adaptive control. The approach combines the SpiNNaker computing platform (Furber and Temple, 2007; Furber et al., 2014), the Neural Engineering Framework (NEF) (Eliasmith and Anderson, 2004) and a series of robots developed at the Technische Universität München. We have previously compared SpiNNaker’s performance when implementing the class of neural networks used by the NEF, showing that it is capable of implementing them ten to twenty times more efficiently than modern CPUs and GPUs (Stewart et al., 2015), even though it was manufactured with what is now a rather old process (130 nm as compared to modern 22 nm or smaller).

The goal of this work is to explore algorithms that can be usefully implemented given hardware that efficiently implements neural networks of this form. In particular, we note that living creatures have both genetic, low-level hard-wired reflexes, and they are also capable of developing novel associations between stimuli and responses that are entirely context dependent. Behavioral studies indicate that they can learn to perform certain actions at certain times, through experience, overriding, and building upon these low-level reflexes (Kim et al., 2007).

However, for applied robotics applications, we do not want an approach where learning is entirely autonomous and undirected [as in, e.g., the neural learning seen in Distributed Adaptive Control (Verschure, 2012)]. Instead, our approach is to inform neural learning by providing explicit indications as to the instances where correct behavior was achieved. The approach described here is somewhat akin to reinforcement learning, but relies only on positive examples, and can be explicitly shaped as desired. This guides the learning and provides explicit control over the eventual behavior.

While the learning system presented here is related to reinforcement learning (Sutton and Barto, 1998), there are important differences. First, the system only requires positive reinforcement (rather than both positive and negative). This both simplifies the model and is reflective of the fact that positive and negative reinforcement are generally considered to be separate systems in living creatures (Boureau and Dayan, 2010). Second, all training is done offline (rather than gradual learning while the robot behavior occurs). Third, the neural connection weights are found by optimization, rather than through a gradient descent learning algorithm. This reduces the catastrophic forgetting problem commonly seen in online learning algorithms.

Our main contribution is a novel method of configuring neural-network-based hardware to perform tasks. This method is a hybrid between explicit programing and autonomous learning, allowing for reliable behavior without the difficulties involved in developing explicit control programs.

## 2 Infrastructure

### 2.1 Embedded Dynamic Vision Sensor: eDVS

The sensor system used here is the eDVS embedded dynamic vision sensor (Conradt et al., 2009), a silicon retina developed by iniLabs in collaboration with the Institute of Neuroinformatics at the University of Zurich and the ETH Zurich. This neuromorphic sensor is a 128 × 128-pixel camera. Instead of reporting frame-based data, it emits individual events when the relative brightness for any individual pixel increases or decreases. The eDVS provides high temporal resolution (~1 *μ*s), low latency (~15 *μ*s), and high dynamic range (~120 dB). The eDVS used here is an embedded version with an onboard microcontroller (NXP LPC4337), inertial measurement unit, multiple PWM control signals, and general-purpose I/O lines. The silicon retina is well suited for integration with other neuromorphic hardware since it produces output in the form of spikes, which is the natural communication framework for spike-based neural processors. Furthermore, certain image processing algorithms can be implemented efficiently. For example, previous work (Müller and Conradt, 2011) has implemented high-speed tracking of flashing LEDs, and we use that algorithm here as sensory pre-processing.

### 2.2 Small Mobile Robot: PushBot

**Figure 1:**
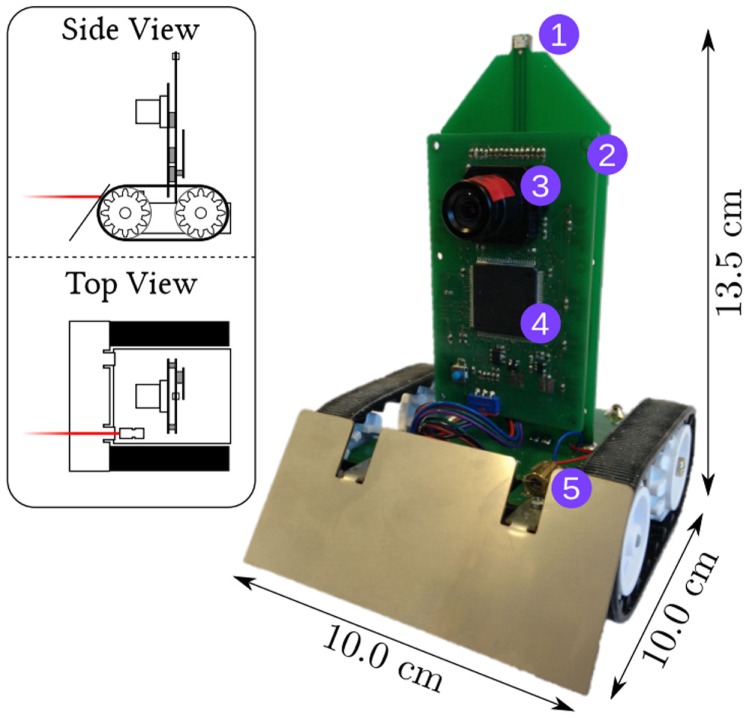
**The PushBot robot with LEDs on the front and back (1), a control board (2) with an eDVS silicon retina (3) and NXP LPC4337 microcontroller (4), and a laser pointer (5)**. The robot communicates through a wireless module on the back (not visible). The top-left insert shows the laser pointer in red.

At the Technische Universität München, the Neuroscientific System Theory group has developed a small tread-based robot built around the eDVS sensor, as shown in Figure 1. The robot provides two motors, a frequency-controllable laser pointer, two LEDs, and housing for power (four AA batteries). The setup comes with a proprietary WLAN module, enabling streaming of sensor data and motor command to and from the robot over standard WiFi to a SpiNNaker hardware interface board (Denk et al., 2013).

### 2.3 Neuromorphic Computing System: SpiNNaker

The SpiNNaker multicore processor is developed by the University of Manchester and consists of 18 200 MHz ARM968 cores on a single die (Furber and Temple, 2007; Furber et al., 2014). The processors and inter-chip communication infrastructure are optimized for massively parallel operations, with a target of a one-million-core machine. Its low power consumption means that a 48-chip SpiNNaker board with a total of 864 processors uses under 40 W of power. This system can be programed directly in C or using the standard neural modeling API PyNN. However, for this work, we made use of the Nengo open-source neural compiler (Bekolay et al., 2014) (described below), and the custom Nengo backend that compiles high-level neural models into optimized SpiNNaker code (Mundy et al., 2015).

For energy-efficient implementation given the hardware, the neuron model employed here is the standard *leaky integrate and fire* (LIF) model, where the weighted (*ω*) sum of input current *I* causes the voltage *V* to build up until some threshold is reached, at which time the neuron emits a discrete spike:

(1) $$\tau_{m}\frac{\mathrm{dV}}{\mathrm{dt}}=-V+\sum \omega I$$

**Figure 2:**
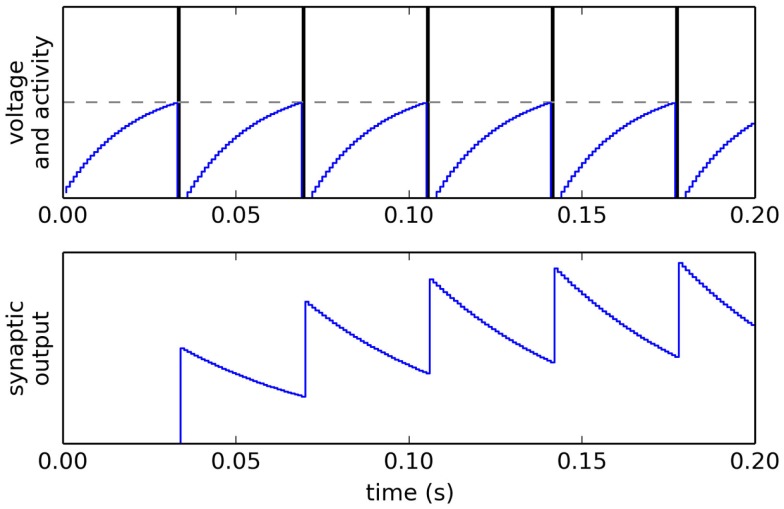
**Voltage *V*, spiking activity and output of a single LIF neuron, given a constant input *I***. SpiNNaker uses a simulation time step of *dt* = 0.001.

The neural membrane time constant (controlling how quickly current leaks out of the neuron) *τ_m_* was fixed at 20 ms. When a spike occurs, this causes post-synaptic current to flow into the neurons to which it is connected, with each connection having its own weight *ω* and with the post-synaptic current *h*(*t*) exponentially decaying over time with a time constant *τ_s_* (fixed at 30 ms), as shown in Figure 2:

(2) $$h\left(t\right)=e^{-t/\tau_{s}}$$

### 2.4 Nengo and the Neural Engineering Framework

The Neural Engineering Framework (NEF) is a general-purpose neural compiler that allows the user to define a high-level algorithm that is then compiled down to a neural approximation of that algorithm (Eliasmith and Anderson, 2004). This approach was originally meant for constructing complex biologically realistic neural models. Demonstrations of the use of Nengo for perception, cognition, and action encoding can be seen in Spaun, the first large-scale (2.5 million neuron) functional brain model capable of performing multiple tasks (Eliasmith et al., 2012).

**Figure 3:**
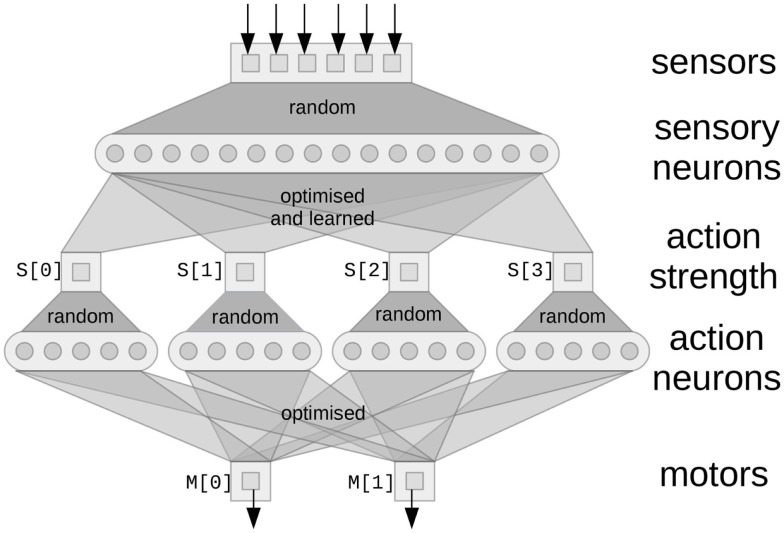
**A network implementing basic reactive control**. Square boxes are the values being represented by the neurons (circles). Random connectivity ensures the neurons form a distributed representation of the vector values that are their input. The optimized output connections are solved for using least-squares minimization to approximate the functions listed in the text. Learned connections are added afterward, as discussed in section 3.2.

The basic concept of modeling using the NEF is the same as most neural network approaches: groups of neurons form distributed representations of their input, and the weighted sum of the output from neurons is used to approximate complex functions. For example, in Figure 3, the six square sensor boxes are the six values returned via the sensory preprocessing on the PushBot robot, indicating the position (*x, y*) and certainty (*c*) information of two flashing lights (the laser pointer dot and the LED on the top of the robot). This information is encoded into the sensory neurons using random connections, which ensures a diverse and rich “hidden layer” representation in the sensory neurons, allowing a wide range of output functions to be represented.^1^

Therefore different patterns of activity within that group of neurons correspond to different vector values being represented. This vector is (normally) of much smaller dimensionality than the number of neurons in the group, so the neural firing forms a redundant code for that vector. Each neuron in the group responds differently to the same input, due to the random input connectivity. Therefore, the redundant code can be thought of as a random projection from a low-dimensional space (the vector being represented) to a high-dimensional space (the neural activity).

Connections out of a group of neurons implement functions on those represented vectors. That is, given some arbitrary function of the input vector, we can find output connections from the neurons that will approximate that desired function. Importantly, due to the redundant code, these functions do not have to be linear functions; rather, any function can be specified, and the system will find the optimal connections between neurons to approximate that function. The Neural Engineering Framework treats this as a least-squares minimization problem and finds the feed-forward linear synaptic connection weights between the individual neurons that will most closely approximate the desired non-linear function. Furthermore, the NEF also indicates how recurrent connections can be found that will approximate any desired differential equation. For both feed-forward and recurrent connections, the accuracy of the approximation will be dependent on the number of neurons and the function being approximated.

As an example, consider a group of neurons storing three values: the *x, y* position of an object and a certainty measure *c* that the object is at that location. This example comes from the output of the tracking algorithm for flashing LEDs (Müller and Conradt, 2011). In the NEF, we might use 100 neurons to form a distributed representation of these three values (using more neurons would improve the accuracy of the representation). Each neuron would be given a random combination of inputs from the three dimensions, resulting in a different pattern of firing for different vectors (*x, y, c*).

Given this activity, we can then define connections to other groups of neurons that compute functions of these values. For example, if we want a group of neurons *R* to store the distance from the center of the visual field to the location of the LED, we could optimize the connections so as to approximate:

(3) $$R\leftarrow \sqrt{x_{\mathrm{LED}}^{2}+y_{\mathrm{LED}}^{2}}$$

This would find synaptic connection weights between the group of neurons representing the LED data and the group of neurons representing the radius *R* such that *R* is driven to fire with whatever pattern represents the correct radius, given the current activity of the LED population.

All of the neural systems described below are defined in this manner, using the software package Nengo (Bekolay et al., 2014) developed specifically for such neural network construction, along with its interface for efficiently simulating such networks on SpiNNaker (Mundy et al., 2015).

## 3 Method

The methodology used here is to start by building a neural system that implements a base set of automatic “reflex-like” behaviors for the robot. This is meant to correspond to the hardwired automatic responses found in living creatures. In addition to this, we will then add a learned system where the robot must map its sensory state to the particular desired actions for this condition. This corresponds to learned associative responses in animals.

### 3.1 Initial Reflexive Control

The first stage of this study requires a definition of the base set of simple behaviors and their triggering conditions, for a small mobile robot. These behaviors should be as simple as possible, since they must be hand designed, but should provide a fairly rich repertoire of resulting actions. These can be thought of as the basic, instinctive reflexes seen in living creatures. In general, the first step in using this methodology is to define a collection of these reflexes.

The first reflexive behavior is simply to go forward if there is no obstruction. To define this, we specify a function that uses sensor data to compute the current strength *S* of an action (i.e., 0–1). In this case, the visual position of the dot caused by the laser pointer mounted on the robot can be used as a simple range detector: if it is lower than some threshold, the robot is near a wall and should not go forward. If it is higher than that threshold, it is safe to go forward:

(4) $$S\left[0\right]\leftarrow \left\{\begin{array}{cc} 1\; & \text{if }y_{\mathrm{LASER}}>-0.6\\ 0\; & \text{otherwise} \end{array}\right.$$

Importantly, the precise threshold of −0.6 is not vital for this model. If the value is slightly higher, then the robot will stop moving forward slightly earlier, but it will still perform qualitatively the same action.

To complete this basic behavior, we define the motor output *M* as a function of *S*. In this case, we want to drive both wheel motors forward when the action has a high strength.

(5) M←[1,1]⋅S[0]

This first reflexive action is depicted in Figure 3 as the left-most square (*S* [0]) and the connections into and out of that square. This action strength will be a scalar value. The connections into that square are optimized using the NEF to best approximate equation (4), given the neural representation of the sensory neurons. To connect this result to the motors (*M*[0] and *M*[1]), it would be possible to directly connect *S*[0] to *M*[0] and *M*[1] with a connection strength of 1. However, this would limit us to only being able to perform *linear* functions. In general, we may want some more complex function to perform the effect of an action, so we perform another random mapping to the action neurons and do another NEF optimization to produce the connections to the motors.

The next basic reflexive action is to back up when we are too close to an obstacle. This is again detected by using the visual location of the dot shown by the laser pointer. If this is very low in the visual field, or if it is not visible at all (i.e., the dot is below the field of view of the camera) then the robot should back up. This gives the following rule:

(6) $$\begin{array}{l} \begin{array}{rl} S\left[1\right] & \leftarrow \left\{\begin{array}{cc} 1\; & \text{if }y_{\mathrm{LASER}}<-0.8\text{ or }c_{\mathrm{LASER}}<0\\ 0\; & \text{otherwise} \end{array}\right.\\ M & \leftarrow \left[-1,-1\right]\cdot S\left[1\right] \end{array} \end{array}$$

The final basic actions are: turn left or right when close to an obstacle.

(7) $$\begin{array}{l} \begin{array}{rl} S\left[2\right] & \leftarrow \left\{\begin{array}{cc} 1\; & \text{if }y_{\mathrm{LASER}}<-0.8\\ 0\; & \text{otherwise} \end{array}\right.\\ M & \leftarrow \left[-1,1\right]\cdot S\left[2\right]\\ S\left[3\right] & \leftarrow \left\{\begin{array}{cc} 1\; & \text{if }y_{\mathrm{LASER}}<-0.8\\ 0\; & \text{otherwise} \end{array}\right.\\ M & \leftarrow \left[1,-1\right]\cdot S\left[3\right] \end{array} \end{array}$$

However, these last two actions should not both be performed at the same time, since this would cause the robot to stay in place, rather than turning one direction or the other. To specify this, we can define connections that relate the strengths of different actions to each other.

(8) $$\begin{array}{l} \begin{array}{r} S\left[2\right]\leftarrow -S\left[3\right]\\ S\left[3\right]\leftarrow -S\left[2\right] \end{array} \end{array}$$

This means that whenever *S*[2] is positive, it will force *S*[3] toward 0, and similarly *S*[3] will drive *S*[2] toward 0. This implements a classic neural competition, meaning that only one of the two actions will have a large value at a particular time.

Now that these reflex actions have been defined in these simple terms, we use Nengo to build neural networks that approximate each of these rules. Groups of neurons are defined to store the sensory state, the motor output, and the strengths of each action. The connections between the groups of neurons are set to approximate each of the above functions, resulting in the neural system shown in Figure 3.

To understand the resulting basic reflexive behavior, two important aspects of NEF must be considered. First, due to the neural representation scheme, when there are multiple inputs to a neural population, the value represented will be the sum of the input values. In other words, the motor output will be the sum of all the connections: *S*[0], *S*[1], *S*[2], and *S*[3] to *M*. Second, the NEF approximations of the desired functions will be *smooth*. That is, instead of implementing the exact functions specified above, the actual behavior will be much more gradual.

**Figure 4:**
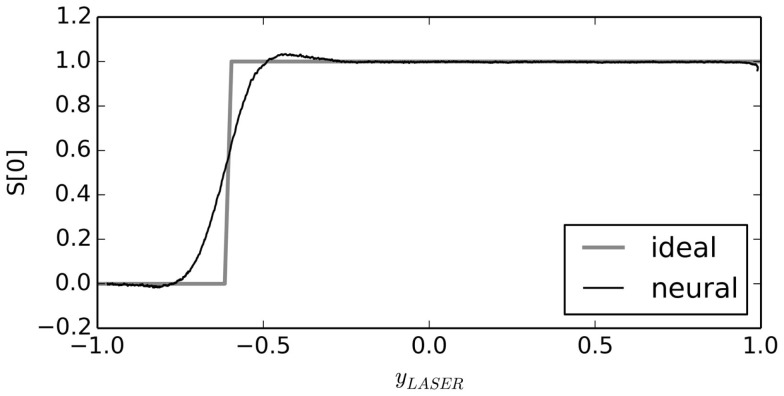
**Neural approximation with the NEF**. When connections between neural groups are optimized to approximate a function, the result is a smooth version of that function. As the number of neurons is increased, this neural approximation will approach the ideal function.

For example, the input function to the *S*[0] population is supposed to be 1 if *y_LASER_* is greater than −0.6, and otherwise it should be 0. Instead of implementing this exact function, the neurons will instead implement a smoothed approximation of this step function, as shown in Figure 4.

The result is a small spiking neural network control system that performs very simple obstacle avoidance. Due to the smoothing inherent in the NEF neural approximation of the above functions, the robot gradually transitions between behaviors. This means that it automatically slows when approaching a wall, even though we have not explicitly built reflexes to do so.

When it reaches a wall, it turns in a random direction. This randomness is due to sensory noise as there is no stochasticity in the neural model itself. Once it has chosen a particular direction to turn, i.e., once either *S*[2] or *S*[3] has a high value, it will continue to turn in that direction until there is no longer an obstacle in front of it. All actions transpire without human interference. The only hardcoded input are the initial thresholds that provide information as to whether an action is, in fact, correct given the current sensory state. These actions demonstrate obstacle avoidance and simple decision making, in much the same way as animal behavior studies (Kim et al., 2007).

The core idea is that this set of behaviors is a bare minimum, sufficient to allow the robot to move throughout its environment without human intervention. As with animal behavior, this allows the robot to have a basic set of automatic decision-making rules that apply in novel situations where there is not yet any learned response. Using these reflexive rules, the robot makes “accidental” decisions (turning left in some situations and turning right in others, for example), which we will then use to further train the robot’s behavior, as discussed in section 3.2.

### 3.2 Serendipitous Offline Learning

While the above approach is sufficient for implementing simple behaviors, it requires the developer to define explicit rules mapping sensory states to actions. In this section, we build upon these rules in a less explicit manner, allowing us to create more complex behavior without hard-coding any additional rules. In complex situations, it may not be feasible for action rules, such as the ones defined above, to be hard coded. Instead, we can also define *implicit* action rules. The basic idea is to allow the robot to explore its environment, but whenever it happens to accidentally do whatever action we want it to do, we record the sensory and motor values and *use those values to define a new rule*.

For example, consider the simple case where we want the robot to always turn left at the intersection, rather than randomly turning either left or right. Recording the sensory data and the strength of each action *S* while the robot is performing its initial basic behavior, we find instances where the robot performs the desired action (turning left). We call these the *positive examples*. In this case, we consider the individual runs where the robot’s rotation happened to remain positive. The data from runs where it turned right are removed. The data from these positive examples can be thought of as long sequences of state-action pairs, indicating what action to perform in what state. Given this, we add new connections from sensors to the action strength populations. Instead of explicitly indicating what functions to approximate on these connections, we instead use the data gathered from the robot itself to be the target function that the neurons must approximate. This adjusts the neural control system, biasing the robot to performing similar actions in similar sensory states.

In order for this to work, there must be some underlying signal in the sensory data that indicates whether or not the action is appropriate. The goal of this learning system is to identify and make use of that signal without requiring explicit programing. To understand what sort of signals this learning mechanism is capable of uncovering, we can evaluate it in an idealized scenario.

**Figure 5:**
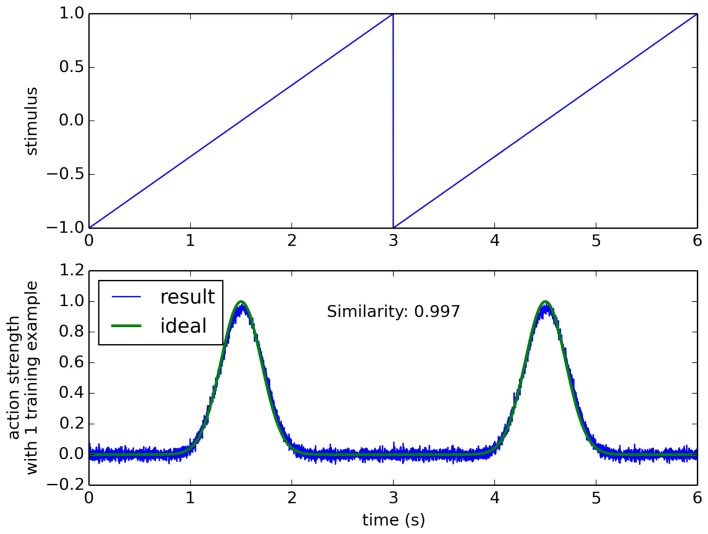
**The learning algorithm applied to a simple synthetic data set**. Sensor data is a ramp (top), and a single positive example is given where the action is performed in the middle of the ramp (bottom “ideal” line). Resulting performance after one training example is 0.997, measured as the normalized dot product between the ideal and the trained result.

For example, in Figure 5 we consider sensory data that is a ramp from −1 to 1 over time, and a desired action that peaks at a particular point on that ramp. When trained from this one positive example, the model is able to successfully trigger that output given the sensory data. We measure the accuracy of this output by computing the similarity between the positive example that it was trained on and the actual neural output, using the normalized dot product. For this simple situation, the model gives a similarity of 0.99. However, the real test is how well such a system will generalize to new conditions, and where there is a more complex relationship between the sensor data and the conditions where the action should be triggered.

**Figure 6:**
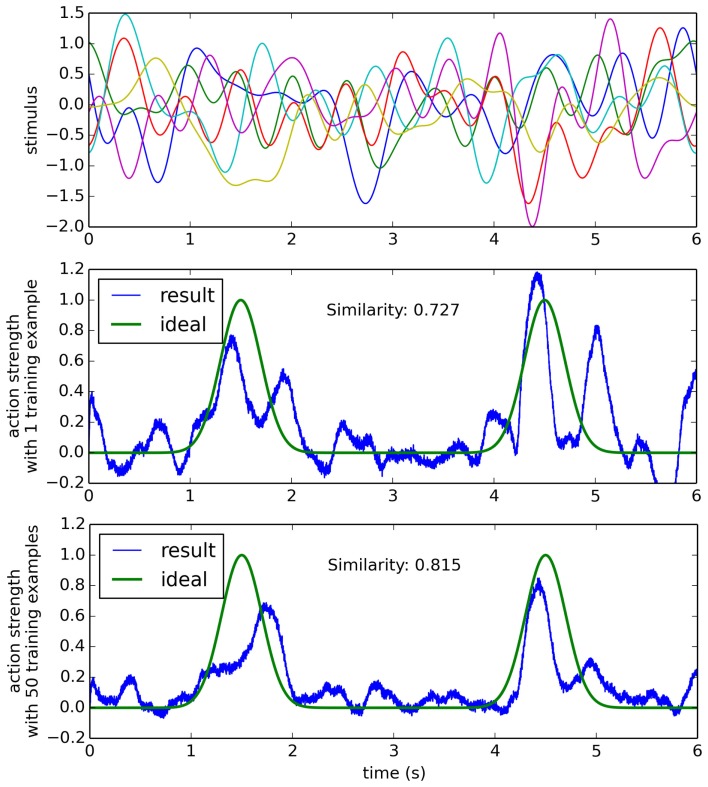
**The learning algorithm applied to a more complex data set**. Sensor data (top) is six-dimensional random gaussian white noise with an added random signal when the action should be performed (see text for details). After a single training example, the result is somewhat correct, but it also performs the action many times when it should not (middle). After 50 training examples, the network is more reliable at performing the action only when it should.

For a more complex situation (but still simpler than the actual robot case), consider a scenario where the input stimulus reflects many different things going on in the environment, but only one of those things is of importance when deciding to perform a particular action. If all of these aspects of the environment add together to create the sensory stimulus, we can model all of the other stimuli to be ignored as a randomly varying signal, plus an additive term that occurs when the action should be performed. That is, the sensory stimulus is *r*(*t*) + *α s*(*t*), where *r*(*t*) is a random N-dimensional band-limited gaussian white noise, *α* is the randomly chosen N-dimensional sensory signal that indicates the situation where the action should be performed, and *s*(*t*) is a scalar that changes over time, adjusting how strong the indication is for this action at different points in time. This situation is shown in Figure 6, and it is clear the learning model performs worse in this condition than in the simpler condition given in Figure 5. In order to understand how our learning model may perform on the physical robot, we can first analyze its behavior in this complex, but abstract scenario.

## 4 Results

### 4.1 Simulated Scenario

Two major parameters which affect the performance of this serendipitous learning algorithm are the strength of the underlying signal and the number of positive examples. The strength of the signal influences how reliable the sensor data is. For a strong signal, the sensor data is a good predictor of whether or not performing the action is the correct thing to do, whereas with a weak signal it is less clear whether the action is correct. As discussed above, we simulate this by generating an artificial sensory stimulus as *r*(*t*) + *α s*(*t*), where |*α*| is the signal strength. The larger |*α*| is the easier it is to predict *s*(*t*) given the sensory stimulus. However, to do this prediction, the learning system also needs to learn to separate *α* from the background *r*(*t*) sensory data that it should ignore. This capability should improve with more positive examples.

**Figure 7:**
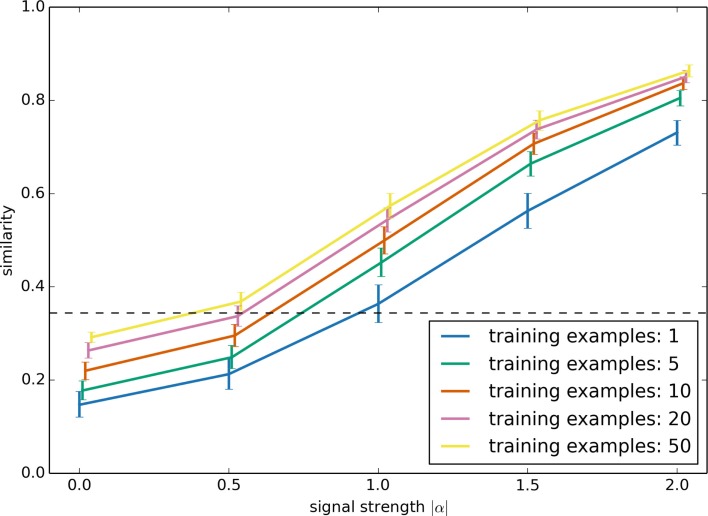
**Model performance as the signal strength |*α*| and the number of positive training examples is varied**. The synthetic data set used here is the same as in Figure 6. Performance is the similarity between the network’s output and the desired ideal output, as measured with the normalized dot product.

These parameters are investigated in Figure 7. In each case, *r*(*t*) and *α* are randomly chosen, and *s*(*t*) is fixed at the pattern shown in Figures 5 and 6. To be consistent with the robot examples below, all values are six dimensional. The training examples had randomly generated *r*(*t*), but with the same *α*.

Performance was evaluated by computing the similarity between the output result of the neural network with the ideal output *s*(*t*). For this, we used the normalized dot product. The result shows that, for cases where there is a clear relationship between sensor data and the action (i.e., for strong signals), this learning system quickly learns to perform the task well, given very few examples (and having more positive examples provides diminishing returns). Error bars are the 95% bootstrap confidence intervals after 150 trials.

The performance given a weak signal is somewhat counter-intuitive. Importantly, note that the system improves its performance with more examples *even if |α| is zero*! That is, even when the sensory stimulus contains no information at all as to whether the action is appropriate, having more examples still improves the model’s performance. This is because the learning system will attempt to predict *s*(*t*) given the sensor data, which in this case is just the randomly chosen *r*(*t*). Given just a few examples, the learning system will make its decision based on spurious correlations between *r*(*t*) and *s*(*t*). In a sense, the model will be *superstitious*. With more examples, this reliance on spurious correlation will be reduced. In the case where *α* is zero, the optimal result is to just output a fixed constant value for *s*(*t*), which can be thought of as the “chance” level performance, indicated with a dashed line in Figure 7.

While this result shows that this learning system should be capable of learning given only a few examples, it also shows that its performance is very dependent on the quality of the sensory data. Determining how this will work in practice requires the use of a physical robot.

### 4.2 Initial Behavior

**Figure 8:**
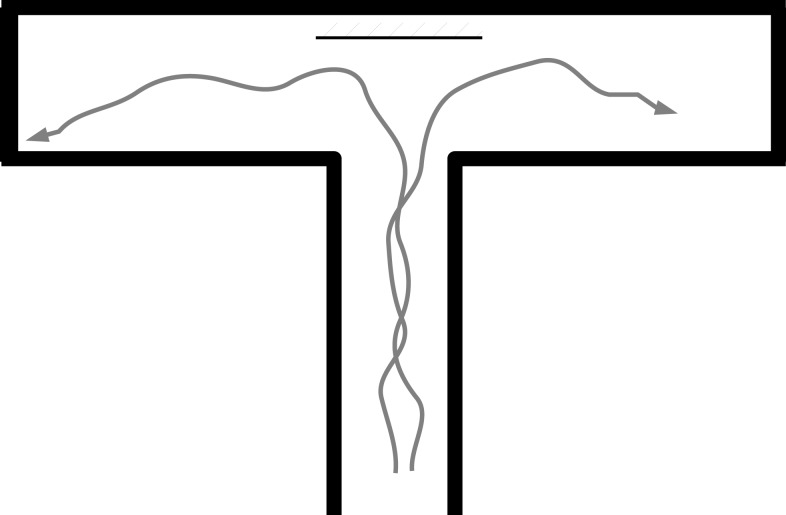
**The T-Maze environment, top-down view**. The robot starts at the bottom of the T shape. A mirror is sometimes placed at the intersection (see section 4.4).

**Figure 9:**
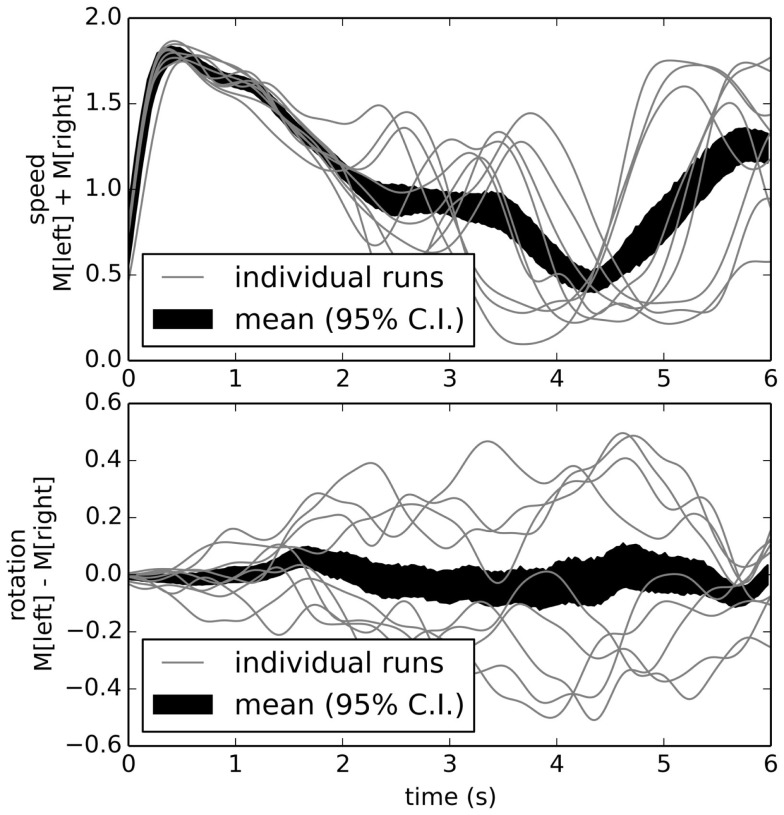
**Behavior of reactive control model over multiple runs**. The speed (top graph) is high at the beginning, then slows as it turns either left or right (bottom graph). While on any individual run the robot tends to turn consistently either left or right, the overall average is zero turning (black area in bottom graph; area is 95% bootstrap confidence interval).

To examine the model’s behavior when run in a physical robot, we used a standard T-maze environment (Figure 8). When placed at the bottom of the T-maze, the robot navigates forward, avoids the walls, reaches the choice point, and turns left or right. Typical trajectories are shown in Figure 9, which indicates the motor outputs over time. Since the robot uses tank-style treads, the two motor output values are the left and right motor speeds, respectively. For clarity, here we plot the overall speed as the sum of M[0] (left motor) and M[1] (right motor) in the upper graph, while the rotation rate is the difference between the two, shown in the lower graph. Motor output values of typical individual runs are plotted along with an overall mean value (with 95% bootstrap confidence interval). All values are Gaussian filtered with *σ* = 0.15 s for visual clarity.

### 4.3 Learning Example 1: Basic Responses

**Figure 10:**
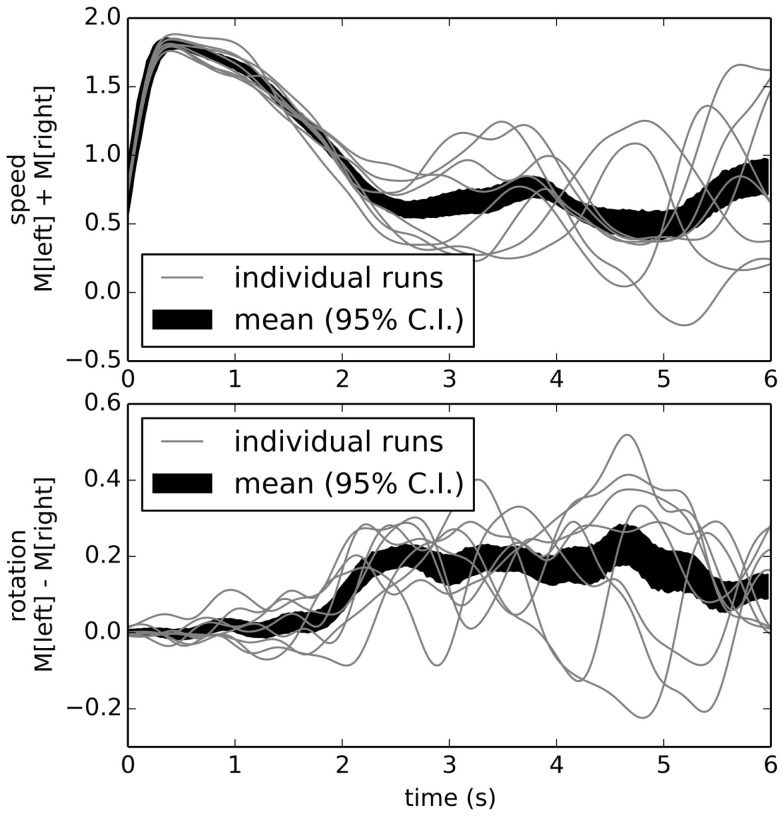
**Behavior after learning to turn left**. By adding connections optimized to approximate situations where the robot behaved appropriately, we implicitly program the robot to map its sensory states to its actions as desired.

For the first and simplest learning task, we chose positive examples where the robot turned left at the intersection. The result of this training after ten positive examples is shown in Figure 10. Unlike Figure 9, this shows the robot consistently turning to the left about 2 s into the task (the average time for the robot to reach the end of the corridor). This indicates we can take a robot with one underlying reflexive control scheme and retrain it to change its behavior without any explicit programing.

### 4.4 Learning Example 2: Sensory Conditions

The initial example of learning to turn left is not particularly complex. However, we can use exactly the same process to produce more complex behavior. To demonstrate this, we now sometimes place a *mirror* at the intersection. Initially, the robot will ignore the mirror and just use its standard random reflexive navigation. We now choose as our positive examples situations where the robot turned right when there was a mirror, and situations where it turned left when there was no mirror.

**Figure 11:**
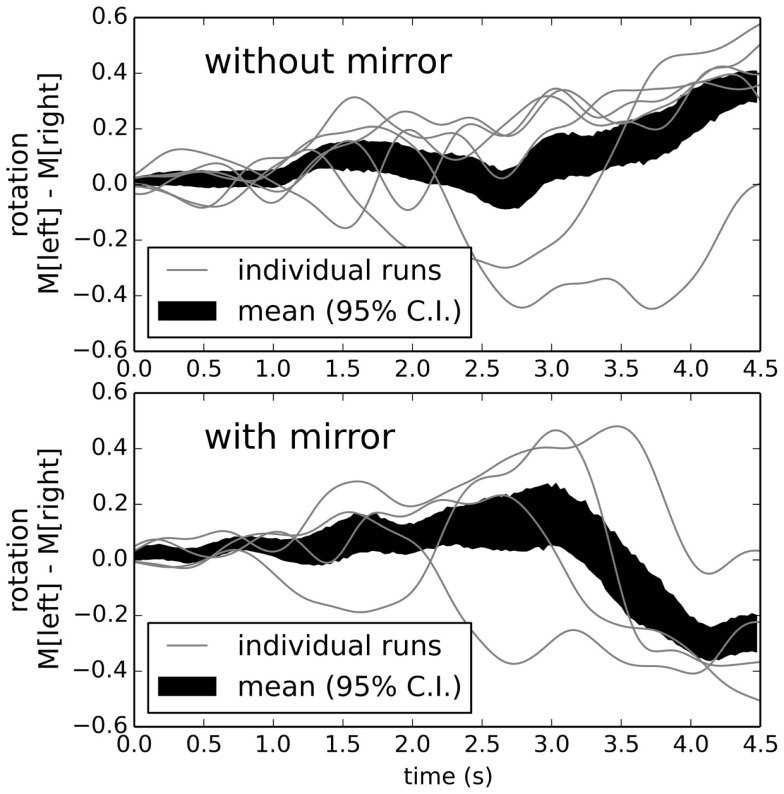
**Behavior after learning to turn right if there is a mirror, and otherwise turn left**. The robot successfully identifies the correct situation and turns appropriately. Robot speed is not shown, but is similar to that depicted at the top of Figure 9.

Adding a learned connection that attempts to approximate those positive examples means that the neural connections now need to change their outputs depending on whether the mirror is present or not. Figure 11 shows that the robot can successfully learn to recognize and respond to mirrors, given only this simple learning rule on top of its basic reflexive rules and ten positive examples.

## 5 Discussion

The algorithm described in this paper is a general-purpose approach to implementing mobile robot behaviors, making use of massively parallel low-power neuromorphic computing hardware. The basic algorithm is as follows:

1. define a set of basic actions (e.g., driving forward, turning left);
2. define functions for the strength of each action, given the current sensory state (e.g., drive forward if there is no obstacle ahead);
3. generate a neural network that approximates these functions;
4. use the neural network to drive the robot and record the neural activity;
5. identify a set of positive examples where the robot has accidentally performed whatever other task is desired (e.g., turning toward a target object);
6. retrain the neural connections between the sensory neurons and the action strengths so as to approximate the data gathered during the positive examples.

Using this approach, we have shown that we can take a network that implements extremely simple manually specified reactive rules, and have it learn by example to perform more complex functions. This method makes use of the power-efficiency of neuromorphic hardware, where large numbers of neurons and connections can be used to efficiently approximate complex functions. Importantly, the functions that the neurons are learning to approximate do not have to be explicitly defined. Instead, we only explicitly define the initial functions for the basic actions. The functions for the more complex actions are implicitly defined *by example*. This means that rather than trying to develop an explicit set of rules the robot should follow, we can instead simply give the robot examples of its own desired behavior. This gives a novel, alternate method for developing robot control.

With any learning method, there is the important question of how well it generalizes and avoids the problem of overfitting. While the fact that the model does perform correctly (Figures 10 and 11) indicates that it has successfully generalized in this case, the simulation results in Figure 7 provide a clearer picture of its performance. In particular, if there is a clear indication in the sensor data as to when the task should be performed (i.e., if the sensor data is unambiguous), then correct generalization performance can be achieved after just a few positive examples. However, if the sensor data is less clear, the learning system will have difficulties. That is, if multiple different conditions in the environment can trigger similar sensory states, the model will have difficulty picking out a sensory pattern that reliably indicates the action should be performed. If this occurs when there are only a few positive examples, then the network will be prone to responding to the *wrong* features of the environment. This can be thought of as the model overfitting to those few positive examples. Fortunately, by increasing the number of positive examples, this overfitting problem is reduced.

While the simulation results indicate good performance for strong signals, it is not yet clear what sorts of tasks provide strong signals. This is a function of the particular sensory data available to the robot, the body morphology, the dynamics of movement, and what the user decides is a positive example. This is a large task space to explore, and this is only a first step. Now that we have demonstrated that this approach is at least possible, we need to rigorously explore these options.

Considering the neural implementation of this algorithm, at first glance, it seems as if the new connections added during the training process (step 6) would just end up being exactly the same as the original reflexive control connections. However, the key difference here is that these new connections will also take into account *other sensory states*, not considered in the original hand-designed reflexive rules. In other words, these new connections will cause the robot to be more likely to perform the actions we desire whenever it is in a sensory state similar to those seen in the positive examples. Importantly, this will happen without any explicit indication of exactly what sensory states should trigger what actions. This allows the system to discover more complex rules than could be reasonably manually hand designed.

In the case of the system learning to change its behavior based on the presence of a reflective mirror surface, the system was able to map the complex sensory stimuli to a particular motor action. In particular, it was able to make use of whatever sensory discrimination is available via the mirror (or lack of a mirror). It is important to note that this sensory stimulus did not initially impact the robot’s behavior in any way. It was only through the process of learning, i.e., using hindsight examples of desired behavior and building a new set of connections that approximate required behavior that triggered changes in movement. This was done by building neural connections that replicate behavior that the robot previously did accidentally.

A vital topic of future work is to characterize the variety of different learned tasks that are amenable to this method. In general, the NEF shows that the accuracy of the learned connections are dependent on the complexity of the function to be learned (in this case the mapping from sensor states to action choices) and the number of neurons (Eliasmith and Anderson, 2004). As we explore different tasks, we will also need to explore different combinations of sensor systems and the pre-processing on this data.

This approach of using sensory experience to learn neural connections that attempt to cause the same actions to occur in response to similar sensory stimuli is novel, but we have performed previous work (Conradt et al., 2014) with a somewhat similar learning method. In that case, we did not have an initial reflexive control system, and we did not find particular examples of desired behavior. Instead, we put the robot temporarily under manual control and used the behavior generated by the person performing the manual control to train the connections between sensory neurons and motor neurons. In other words, rather than allowing the robot to randomly explore its environment and choose actions based on a manually created reflexive control system, we had a user manually set the motor outputs using a remote control. As in this work presented here, we then trained neural connections that would approximate that manual control.

In that earlier work, learning required direct training examples, rather than simply labeling particular actions as positive example of desired behavior after they occur. Indeed, this means that the work presented here could be thought of as reinforcement learning, but the previous work would be purely supervised learning. Of course, a hybrid approach could be pursued.

Finally, it should be noted that we are only training based on positive examples (i.e., situations where the robot behaved as desired). In future work, we plan to augment this approach with explicit punishment for situations where the robot performs a non-desired behavior. Interestingly, adding this capability is not straight-forward in this case. Indeed, there is significant biological evidence that positive (reward) and negative (punishment) systems are separate in the brain (Boureau and Dayan, 2010). That is, they are not simply the opposite of each other – rather they are significantly different processes that interact. This interaction is still to be explored. One step toward, this is a model of fear conditioning (Kolbeck et al., 2013), which would fit well with this framework.

## Author Contributions

TS has designed and performed experiments (modeling in Nengo), recorded data, and evaluated results. AM has developed the software interface between Nengo and SpiNNaker, revised models in Nengo, and assisted in experiments, data collection, and evaluation. AK has designed and performed robotic experiments, implemented models in Nengo, and analyzed results. JC has developed and build the robot and hardware infrastructure (SpiNNaker interface boards and communication), revised experiments, and evaluated results. All four authors have contributed in the design of the experiment, in the evaluation of data, and in writing the paper.

## Conflict of Interest Statement

The authors declare that the research was conducted in the absence of any commercial or financial relationships that could be construed as a potential conflict of interest.

## Footnotes

^1^One can use a learning rule such as backpropagation of error to optimize these weights, but we generally find this to be unnecessary
